# Organic Nitrates and Nitrate Resistance in Diabetes: The Role of Vascular Dysfunction and Oxidative Stress with Emphasis on Antioxidant Properties of Pentaerithrityl Tetranitrate

**DOI:** 10.1155/2010/213176

**Published:** 2010-12-27

**Authors:** Matthias Oelze, Swenja Schuhmacher, Andreas Daiber

**Affiliations:** The 2nd Medical Clinic, Laboratory for Molecular Cardiology, University Medical Center, Johannes Gutenberg University of Mainz, 55101 Mainz, Germany

## Abstract

Organic nitrates represent a class of drugs which are clinically used for treatment of ischemic symptoms of angina as well as for congestive heart failure based on the idea to overcome the impaired NO bioavailability by “NO” replacement therapy. The present paper is focused on parallels between diabetes mellitus and nitrate tolerance, and aims to discuss the mechanisms underlying nitrate resistance in the setting of diabetes. Since oxidative stress was identified as an important factor in the development of tolerance to organic nitrates, but also represents a hallmark of diabetic complications, this may represent a common principle for both disorders where therapeutic intervention should start. This paper examines the evidence supporting the hypothesis that pentaerithrityl tetranitrate may represent a nitrate for treatment of ischemia in diabetic patients. This evidence is based on the considerations of parallels between diabetes mellitus and nitrate tolerance as well as on preliminary data from experimental diabetes studies.

## 1. Introduction

### 1.1. Clinical and Economical Importance of Diabetes Mellitus

According to an estimation of the World Health Organization (WHO) in November 2009, more than 220 million people worldwide suffer from diabetes mellitus, a figure that will probably double by the year 2030. The economic and human costs of this disease are devastating. WHO estimates that in the period 2006–2015, China will lose $558 billion in foregone national income due to heart disease, stroke, and diabetes alone. The total cost of diabetes in the United States in 2002 was $132 billion. According to the statistics of the NIH in 2007, 23 million Americans had diabetes, and the estimated lifetime risk for Americans born in 2000 to develop diabetes is 1 in 3. In adults, diabetes is the most common cause of blindness, nontraumatic amputations, and end-stage renal disease as well as the sixth most common cause of death. In Germany, every 19 minutes a person with diabetes suffers a heart attack. With regard to these facts, it is of high clinical and economical importance, but also in the interest of the patients and their quality of life, to find new therapeutic options for the treatment of diabetes. Primarily, this research should be focused on the treatment of the metabolic disorder underlying diabetes to stop or slow down its progression. But also the treatment of diabetes symptoms and complications need to be addressed to improve the quality of life and prognosis of diabetic patients. Yet only limited knowledge exists about the pathogenesis, the cardiovascular consequences, and the prevention of this disease [1]. Recent data suggest that the cardiovascular complications in diabetes mellitus are associated with oxidative stress [2, 3], as previously shown for hypercholesterolemia and arteriosclerosis [4, 5]. The sources of these reactive oxygen and nitrogen species (RONS) have been identified to be NADPH oxidases, mitochondria, xanthine oxidase, as well as an uncoupled endothelial nitric oxide synthase (eNOS) [6–8]. The present review will address the question whether organic nitrates may be used for treatment of diabetic patients, and if there are differences between the organic nitrates being in clinical use at present.

### 1.2. Biochemical and Metabolic Consequences of Diabetes Mellitus

**Figure 1:**
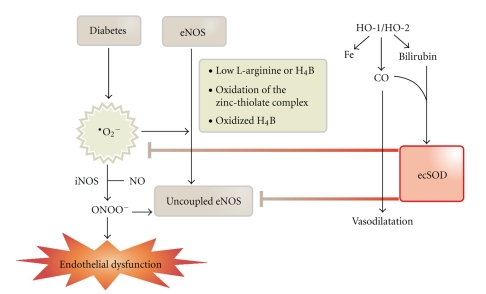
The scheme summarizes the simplified mechanisms underlying oxidative stress-induced endothelial dysfunction (and probably nitrate resistance) in diabetes mellitus. It should be noted that the oxidative stress concept provides an explanation for a part of diabetic complications and probably represents one important pathological pathway among several. Prevention of diabetic cardiovascular complications by induction of the heme oxygenase antioxidant system. Key mediators of these beneficial effects are carbon monoxide (CO) bilirubin, extracellular superoxide dismutase (ecSOD), coupling of endothelial NO synthase (eNOS) by normalization of tetrahydrobiopterin (H_4_B) levels, and decrease in superoxide levels. Adopted from Abraham and Kappas, *Pharmacol. Rev.* 2008 [31].

**Figure 2:**
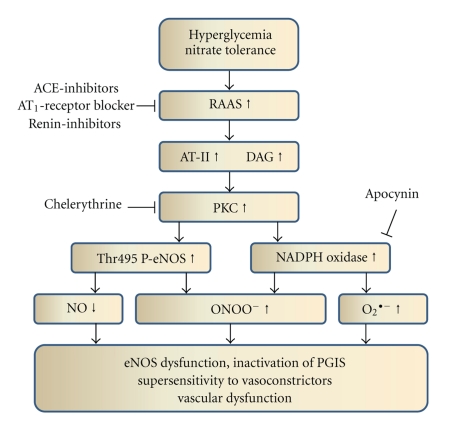
Sequence of events during activation of the renin-angiotensin-aldosterone system (RAAS) in the setting of hyperglycemia (diabetes mellitus type 1 and 2) or nitrate tolerance. Some therapeutic and experimental interventions are also shown. AT-II: angiotensin-II; DAG: diacylglycerol; PKC: protein kinase C; Thr495 P-eNOS: threonine495-phosphorylated endothelial NO synthase; NO: nitric oxide; O_2_^•−^: superoxide; ONOO^−^: peroxynitrite; PGIS: prostacyclin.

The relationship between diabetes and the resulting cardiovascular complications is complex, but damage to the vascular endothelium obviously plays a major role [9]. Chronic exposure of proteins from plasma and the cell membrane to hyperglycemia leads to the attachment of glucose molecules to proteins, a process known as nonenzymatic glycosylation. Subsequent slow reactions cause the formation of advanced glycation end products (AGEs) [10] known to inactivate nitric oxide (NO) and impair endothelium-dependent vasodilation [11]. However, short-term hyperglycemia leads to similar negative effects on vascular responses, suggesting that this is not the only mechanism [12]. It was shown that hyperglycemia inhibits eNOS-dependent NO formation in favour of superoxide production, leading to an impaired endothelium-dependent vasodilation [13]. Besides the formation of advanced glycation end-products and diacylglycerol (DAG), the simultaneous increase in production of reactive oxygen species (especially superoxide, O_2_^•−^) contributes to inhibition and uncoupling of eNOS via depletion of BH_4_ (summarized in Figure 1). Moreover, in diabetic patients, a higher production of vasoconstrictive compounds, such as endothelin-1 and angiotensin-II, has been observed. The increase in DAG and angiotensin-II is a direct consequence of the activation of the renin-angiotensin-aldosterone system (RAAS) in the setting of diabetes [14], which is also closely related to endothelial dysfunction [15, 16]. Since DAG is a potent activator of the protein kinase C [17, 18] with subsequent activation of the NADPH oxidase [19], RAAS-induced activation of this superoxide source was demonstrated by clinical [20] as well as animal experimental studies [21]. There is even some evidence that suppression of the RAAS is able to prevent the development or progression of diabetes mellitus type II in hypertensive patients [22]. The events taking place during activation of the RAAS are summarized in Figure 2.

Leukocyte infiltration into the vascular wall was also dramatically increased. The increased concentrations of cytokines and matrix metalloproteinases lead to a decreased synthesis and increased degradation of collagen in atherosclerotic plaques reducing the stability of the fibrous cover plate, finally causing plaque rupture, thrombus formation, and myocardial infarction. In addition, the thrombocytes in diabetic individuals are larger in size, express more glycoprotein receptors on the surface, and show more pronounced aggregation in response to different stimuli (for review see [23]). Patients with diabetes mellitus have a 2- to 4-fold increased risk for infarction, and the mechanistic basis for macrovascular complications is progression of atherosclerosis. Endothelial dysfunction is a hallmark in the early stage of diabetes mellitus type 1 and 2 [24]. Oxidative protein modifications such as 3-nitrotyrosine formation [25], oxidative disruption of the zinc-sulphur-cluster, and uncoupling of eNOS [26] as well as increased levels of toxic aldehydes [27] are common features of diabetes and may contribute to the observed disorders. A broad variety of different antioxidants have been demonstrated to beneficially influence diabetic complications [27–30].

## 2. Organic Nitrates

### 2.1. Clinical Use of Organic Nitrates

Nitroglycerin (glyceryl trinitrate) and the long-acting nitrates (isosorbide-5-mononitrate [ISMN], isosorbide dinitrate [ISDN], and pentaerithrityl tetranitrate [PETN]) have been used in cardiovascular medicine for >100 years. Despite the fact that the potential mechanisms for relief of myocardial ischemia with nitrates are multiple and not yet fully understood [32], nitrates are widely utilized for the various anginal syndromes, and are also used in congestive heart failure and in patients with left ventricular dysfunction [33, 34]. The nitrovasodilators are a group of drugs that result in the formation of NO or a related species within vascular smooth muscle cells [35]. Following the discovery of endothelium-derived NO [36], the idea of NO being the active principle of nitrates was very attractive to the scientific community, and it led to the speculation that they may replace a compromised endothelial NO production [37], such as in patients with coronary heart disease. Nitrates also have antiaggregatory effects, and recent evidence confirms that these drugs decrease platelet aggregation and thrombosis formation [38], although for nitroglycerin, an activation of thrombocytes was described [39, 40]. This may play an important role in the therapy of acute unstable myocardial ischemia, including unstable angina and myocardial infarction. The hemodynamic effects of nitrates were known for a long time. They are primarily modulated by decreased myocardial work that results from smaller cardiac chambers that operate with lower systolic and diastolic pressures. These changes are secondary to a redistribution of the circulating blood volume from cardiac tissue to the venous capacitance system, with a decrease in venous return to the heart. The effects of nitrates on afterload as well as on arteries are also useful for the decrease in myocardial oxygen consumption. Considerable evidence supports a variety of mechanisms whereby nitrates lead to increased coronary blood flow, including stenosis enlargement, epicardial coronary artery dilation, improvement of endothelial dysfunction, enhanced collateral size and flow, and prevention or reversal of coronary artery vasoconstriction [33].

### 2.2. Bioactivation of Organic Nitrates

There is a consensus that the principle mechanism of organic nitrate-induced smooth muscle relaxation is achieved via NO signalling pathways, including activation of soluble guanylyl cyclase, increase in cyclic GMP levels and activation of cyclic GMP-dependent protein kinases, and/or cyclic nucleotide-gated ion channels which was extensively reviewed [32]. Recently, the organic nitrate-derived NO hypothesis was challenged by two independent studies using state-of-the-art-methods to detect nitrate-derived NO formation which showed a huge discrepancy between nitroglycerin-derived NO and NO-induced vasodilation [35, 41]. This, however, questions the relevance of nitroglycerin-derived NO formation in the clinically relevant concentration range whereas NO was detected for ISMN and ISDN during vasodilation of isolated aortic rings. For nitroglycerin, several bioactivating systems have been proposed during the last decades including xanthine oxidoreductase, glutathione-S-transferases, and cytochrome P450 enzymes (for review see [32, 34]). It should be noted that these reports on nitroglycerin-derived NO formation were based on high suprapharmacological concentrations (>10 *μ*M) of the nitrate. Mononitrates and dinitrates have been reported to be mainly bioactivated by cytochrome P450 enzymes. In 2002, the mitochondrial aldehyde dehydrogenase (ALDH-2) was identified as an organic nitrate bioactivating enzyme [42]. This so-called nitrate reductase activity denitrates nitroglycerin to its 1,2-glycerol dinitrate metabolite and nitrite. This reaction relies on reduced thiols at the active site of the enzyme and on the presence of reduced dithiols as the electron source. During bioconversion of nitroglycerin, but also in the presence of reactive oxygen and nitrogen species, the active site thiols of ALDH-2 are oxidized, and the enzyme loses its activity [43]. Interestingly, ALDH-2 bioactivates nitroglycerin, PETN, and its trinitrate metabolite PETriN but not ISMN and ISDN [44], which could explain the different potency of these nitrates.

### 2.3. Clinical Tolerance, Endothelial Dysfunction, and Differences between Organic Nitrates

Tolerance and cross-tolerance to endothelium-dependent vasodilators (endothelial dysfunction) are a major limitation for chronic nitrate treatment. Clinical tolerance and cross-tolerance may involve counterregulatory mechanisms (pseudotolerance) including release of catecholamines and activation of the renin-angiotensin-aldosterone system (RAAS) making these phenomena even more complicated [32]. The first evidence for an involvement of the renin-angiotensin-aldosterone-system (RAAS) in GTN-induced nitrate tolerance came from observations that long-term inhibition of angiotensin-converting enzyme improves nitrate tolerance in dogs [45, 46]. Additional studies showed that AT_1_-receptor blockade and ACE inhibition positively influence the development of tolerance, and it was hypothesized that this protective effect is mediated via inhibition of NADPH oxidase-dependent ROS formation [45, 47–53]. In contrast, there is a limited number of studies reporting on lack of effect of ACE inhibitors and AT_1_-receptor blockers on nitrate tolerance [54–56] and nitrate-induced oxidative stress [57]. Downstream to the RAAS system is the protein kinase C (PKC), which was also identified as an important constituent in the development and maintenance of GTN-induced nitrate tolerance [58–60]. Moreover, it was demonstrated that GTN-induced activation of PKC mediates late preconditioning, a protective effect which is probably based on initial ROS formation and mild oxidative damage [61]. The contribution of NAD(P)H oxidases to ROS production and oxidative damage in response to GTN therapy was established by a number of independent studies [62–64]. Nevertheless, NADPH oxidase, as a source of ROS formation in the setting of tolerance, was questioned since DPI, an unspecific inhibitor of flavin-dependent oxidoreductases, could not prevent the impaired response to GTN in tolerant vessels *ex vivo* [65]. In summary, the majority of previous work supports the contribution of the RAAS, PKC, and NADPH oxidase to the development of nitrate tolerance (the sequence of events is provided in Figure 2) although some studies neglect the importance of this pathway.

**Figure 3:**
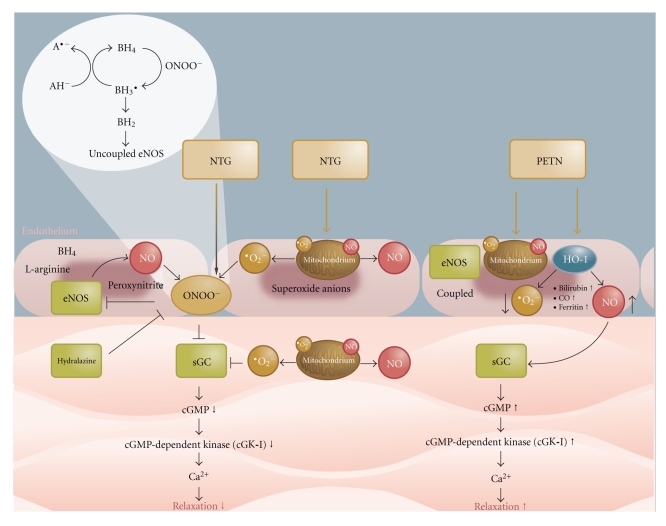
Vasodilation by nitroglycerin (NTG) and PETN: bioactivation to NO or a related species with subsequent activation of soluble guanylyl cyclase (sGC) and smooth muscle relaxation. Mechanisms underlying induction of vascular dysfunction by nitroglycerin and prevention of these adverse effects by activation of antioxidant, protective pathways by PETN: nitroglycerin induces mitochondrial and NADPH oxidase-dependent oxidative stress leading to uncoupling of eNOS and desensitization of sGC. PETN prevents these harmful events by induction of the antioxidant principle heme oxygenase and ferritin as well as others which are not shown here. Adopted from Gori et al., *Arzneimitteltherapie* 2008 [81].

Tolerance and/or endothelial dysfunction have been observed in response to chronic treatment with nitroglycerin [66, 67], ISMN [68], and ISDN [69] (studies before 1990 reviewed in [70]) whereas no tolerance phenomena or markers such as oxidative stress were observed for long-term administration of PETN [67, 71]. These reports indicate that tolerance development is uniform to all organic nitrates if applied in clinically effective dosage for longer periods of time. A remarkable exception seems to be PETN [72]. Some of these effects are related to special pharmacokinetics of PETN [73, 74], but, upon chronic administration, PETN also induces antioxidant pathways at the genomic level and cardioprotective genes (e.g., Apex1 and NFAT5) [75]. Among these induced antioxidant genes are heme oxygenase-1 (HO-1) and ferritin [76], both possessing highly protective properties. There is good experimental evidence that at least part of the beneficial profile of long-term PETN treatment is based on activation of the heme oxygenase-1/ferritin system [77] and preliminary data in heme oxygenase-1 knockout mice support these previous findings. According to recent, unpublished data from our laboratory, PETN even acts differently at the purified ALDH-2, preventing inactivation of this nitrate reductase. Since ALDH-2, beyond bioactivating organic nitrates, was recently identified as an important protective enzyme, preventing ischemic damage in experimental myocardial infarction [78], the conservation of the enzymatic activity of ALDH-2 by an organic nitrate may be of great importance for long-term treatment of patients with ischemic heart disease. Nitroglycerin not only inactivates ALDH-2 but also downregulates the enzyme at the protein level [79]. More differences between organic nitrates in clinical use were recently discussed in detail [80]. The differences between nitroglycerin and PETN are summarized in Figure 3.

### 2.4. Nitrate Tolerance and the “Oxidative Stress Concept”

The “oxidative stress concept” in the setting of nitrate tolerance was established by Munzel and colleagues [82] and refined during the last couple of years [66, 83]. In essence, the concept consists of increased superoxide formation in response to nitrate treatment which decreases NO bioavailability, leads to peroxynitrite formation, NOS uncoupling, and impairs NO/cyclic GMP signalling [66]. Moreover, oxidative inhibition of prostacyclin synthase [84] as well as mitochondrial ALDH activity [85] may present other key events in the development of nitrate tolerance. Previously, the vascular NADH oxidase was considered as the most important source of reactive oxygen species (ROS) in the setting of nitrate tolerance [32]. Today this concept has been extended to induction of mitochondrial oxidative stress by nitroglycerin treatment [83, 86] and a crosstalk between mitochondrial ROS with NADPH oxidases in the cytoplasmic membrane [87]. Induction of oxidative stress was also described for ISMN in experimental hypertension [88], and indirect evidence was presented recently by demonstrating that the antioxidant vitamin C reverses ISMN-induced endothelial dysfunction in healthy volunteers [68]. However, until now, there is no direct proof for induction of oxidative stress by ISMN from clinical trials [89]. For ISDN, increased activation of NADPH oxidase was demonstrated in experimental MI as well as the adverse effects of the nitrate on the function of endothelial progenitor cells [90] although previous work by Keimer et al. revealed no induction of oxidative stress under ISDN or PETN therapy in healthy volunteers [91]. Indirect evidence for a role of oxidative stress in ISDN therapy comes from the highly beneficial effects of hydralazine combination therapy [92] since hydralazine is a powerful antioxidant [93] and has been demonstrated to prevent nitroglycerin-induced tolerance [62]. Related to these observations on adverse effects of nitrate therapy, Nakamura et al. presented evidence that long-term nitrate therapy increases cardiovascular mortality based on a retrospective analysis using databases from two large-scale postinfarction studies [94]. It should be noted that this analysis did not distinguish between different nitrates although there is increasing evidence that organic nitrates should not be treated as a homogenous class of compounds. Moreover, it is important to keep in mind that PETN did not share the adverse effects of the other nitrates since it was devoid of tolerance [67, 71, 74], induction of oxidative stress [71, 90] and downregulation of protective genes [75, 77]. Recent data has shown that nitroglycerin and PETN, although both considered as NO donors, exert completely opposite effects with respect to gene regulation: PETN in vivo treatment induced cardioprotective genes (e.g., Apex1 and NFAT5) whereas nitroglycerin in vivo administration induced cardiotoxic genes (e.g., Egr1, fos, junD, and MITF) [75]. Another highly important property of PETN may be the induction of the extracellular superoxide dismutase (ecSOD) [95] and glutathione peroxidase (GPx) [96], two highly protective antioxidant proteins. These experimental and clinical data on the beneficial effects of PETN were recently supported by the double-blind, placebo-controlled PENTA study conducted by Warnholtz and colleagues with the main finding that chronic PETN treatment does not induce endothelial dysfunction or nitrate tolerance, but even improves GTN potency [97]. Relative changes in mean flow volume and mean flow velocity upon ischemia increased in the PETN group versus controls.

## 3. Use of Organic Nitrates for Therapy of Diabetic Patients

### 3.1. Nitrate Resistance and Diabetes

The endothelial dysfunction present in hypercholesterolemic, type 2 diabetic, smoking, and ischemic heart disease patients reflects the reduced nitric oxide bioavailability, which can be assessed by different methods [98]. Patients with chronic heart failure did not show a significant effect in response to the NOS inhibitor N^G^-methyl-L-arginine suggesting that NO does not contribute to basal vascular tone in these individuals. The responses to nitroglycerin and to serotonin were impaired, suggesting that there is smooth muscle dysfunction and endothelial dysfunction in patients with chronic heart failure [99]. Nitrate tolerance can be seen as an extreme example of nitrate resistance [100], and is thought, at least in part, to be secondary to the increased oxidative stress present in the aforementioned disease states. Thus, clinical nitrate tolerance and nitrate-induced activation of the renin-angiotensin-aldosterone system (RAAS) as well as increased oxidant stress (see Section 2.3) may be regarded as an extension of the primary pathophysiological phenomenon of nitric oxide resistance (see Figure 2) [100]. Since diabetes is also associated with severe activation of the RAAS (see Section 1.2), the effects of diabetes and nitrate therapy could be additive and further impair endothelial function as well as nitrate potency. Hence, the potency of nitrates may be blunted by an underlying primary NO resistance to give a “primary” nitrate tolerance [101] whereas the mechanistically similar “secondary” nitrate tolerance (pseudotolerance) develops under chronic treatment [102]. These two nitrate tolerance states can even occur within the same patient. However, to date, it is not completely clarified whether pseudotolerance (secondary nitrate tolerance) contributes to endothelial dysfunction in response to an impaired NO responsiveness (NO resistance). However, evidence supporting this notion comes from a multivariate analysis of two observational coronary secondary prevention studies which showed increased mortality in chronically nitrate-treated ischemic heart disease patients following recovery from an acute cardiac event [94]. Also, a randomized controlled trial was performed and showed that continuous nitroglycerin treatment leads to endothelial dysfunction in both ischemic heart disease patients [103] and healthy volunteers [104]. In a series of experiments, impaired vasodilator responses to nitroglycerin were demonstrated in patients with diabetes mellitus when compared with age and sex matched controls [105, 106].

These impaired responses were somewhat surprising since nitrate-induced vasodilation has been shown to be increased when the endothelium is damaged and diabetes is associated with impaired endothelial function [9]. A possible explanation may be that the biotransformation of organic nitrates requires intracellular sulfhydryl groups in order to produce vasoactive intermediates [107]. Oxidative depletion of these sulfhydryl donors will cause impaired responsiveness to organic nitrates. In diabetes antioxidant activity is decreased [108]. The increase in oxidative stress and free radical activity that occurs in diabetes probably alters the redox equilibrium of intracellular thiols leading to primary oxidation or depletion of these essential sulfhydryl donors. But also succination of thiols may contribute to decreased sulfhydryl concentration [109]. Depletion of thiols is also envisaged in rats with streptozotocin-induced experimental diabetes that have significantly decreased serum antioxidant capacity (which largely depends on serum thiol groups) (unpublished observations, Oelze and Daiber). This is one possible explanation for an impaired nitrate response observed in diabetes [102, 105]. According to the recent development in the nitrate field, the mitochondrial nitrate reductase ALDH-2 could play a role as well. This enzyme requires dithiols (e.g., dihydrolipoic acid) in order to bioactivate nitroglycerin or PETN [43] and depletion of the pool of reduced thiols would probably lead to impairment of enzymatic activity. Although PETN also requires dithiols for bioactivation, this organic nitrate has slow pharmacokinetics preventing an overcharge of the bioactivating capacity of the organism [74, 110]. This hypothesis is in accordance with the recent observation that ALDH-2 activity is decreased in the testis of diabetic animals [111] although this observations needs further animal experimental or clinical verification in vascular tissue. Vice versa, inactivation of ALDH-2 by nitroglycerin was demonstrated to increase the infarct area in experimental MI [78] and ALDH-2 activation is related to PKC*ε* [112]. Recently, Ma et al. have demonstrated that ALDH-2 knockout aggravated, and ALDH-2 overexpression improved, ischemic damage in response to experimental MI [113]. Interestingly, these authors revealed a correlation between ALDH-2 and AMP-activated kinase (AMPK), which is an important metabolic enzyme involved in the management of disease states such as diabetes [114–116]. Besides the loss of AMPK activity by GTN-induced inactivation of ALDH-2, diabetic complications could be further aggravated by impaired breakdown of toxic aldehydes—it may be speculated that inhibition of ALDH-2 and AMPK may contribute to increased mortality of diabetic patients. In conclusion, for patients with congestive cardiac failure, type II diabetes and acute myocardial infarction resistance to the action of organic nitrates was demonstrated. In addition, there is good evidence that a significant percentage of normal subjects have impaired organic nitrate vasodilator potency [117].

### 3.2. Effectiveness of Antioxidants in Diabetes- and Nitrate-Associated Vascular Complications

It was previously shown for the setting of nitrate tolerance and diabetes that there is increased formation of reactive oxygen species and/or decreased breakdown of those species leading to oxidative stress [2, 3, 66, 82]. Accordingly, there is a large number of studies demonstrating that cotreatment with antioxidants ameliorates nitrate- and diabetes-induced vascular oxidative stress and dysfunction. Examples include the use of direct antioxidants such as hydralazine [62, 93], vitamin C [40], superoxide dismutase [82], N-acetylcysteine [118], ebselen [84], and drugs with indirect antioxidant properties such as statins [119], AT_1_-receptor antagonists [59], ACE inhibitors [52], and the *β*-blocker carvedilol [120] (more compounds are reviewed in [32]). The antioxidant lipoic acid could improve nitrate tolerance which could be partially due to the fact that lipoic acid is the reducing cofactor of the nitrate bioactivating enzyme ALDH-2 [43, 74, 121]. It should be also noted that NO itself is a highly potent superoxide scavenger, although the resulting formation of peroxynitrite may limit the importance of this antioxidant property. Accordingly, organic nitrates with high turnover to NO may provide intrinsic antioxidant effects. The important role of oxidative stress for the development and progression of nitrate tolerance was further supported at a molecular level by the observation that vascular dysfunction in response to nitroglycerin was improved by genetic deletion of p47^phox^, an essential subunit of the superoxide generating NADPH oxidase (Nox2) [87]. In contrast, genetic deletion of the important mitochondrial antioxidant protein manganese superoxide dismutase (MnSOD) caused an additional aggravation of nitrate-induced vascular dysfunction [83]. Interestingly, diabetes shows a very similar responsiveness to antioxidant treatment indicating that similar mechanisms as in nitrate tolerance account for vascular complications in diabetic patients and animals (see the aforementioned triggers for nitrate resistance and endothelial dysfunction). Improvement of vascular dysfunction and oxidative stress in diabetes was shown for direct antioxidants such as vitamin C [122, 123], lipoic acid [28, 124], ebselen [25], N-acetylcysteine [125], glutathione [126], and drugs with indirect antioxidant properties such as statins [127], AT_1_-receptor antagonists, or ACE inhibitors [8, 21, 128]. It should be noted that vitamin C was infused at high doses whereas oral administration did not improve endothelial dysfunction in diabetic patients [129] and even increased mortality in diabetic women [130]. Further evidence for an important role of oxidative stress for diabetic complications was provided at a molecular level by demonstrating that oxidative modifications contributing to diabetic retinopathy were prevented by overexpression of the MnSOD in a transgenic mouse model [131]. Likewise cardiomyocyte function and oxidative stress were beneficially influenced by overexpression of catalase in experimental models of type 1 and 2 diabetes [29]. Based on these data, there are striking parallels between nitrate tolerance and diabetes mellitus that converge at the level of oxidative stress as well as vascular dysfunction, which may explain nitrate resistance in the setting of diabetes. In accordance with these considerations, the clinical study of Picano and coworkers demonstrated that diabetes and nitrate therapy evoke synergistic oxidative DNA damage [132] suggesting that the pathophysiological mechanisms are similar in the setting of diabetes and nitrate tolerance. Comparable observations were made in atherosclerotic rabbits where nitroglycerin treatment synergistically increased the protein tyrosine nitration (a marker for peroxynitrite formation) in hyperlipidemic animals [133].

### 3.3. Is Diabetes Mellitus Another Battlefield for Pentaerithrityl Tetranitrate?

As discussed above, the use of organic nitrates in diabetic patients is associated with certain risks: besides reduced efficacy of the drugs due to nitrate resistance in these individuals, there might be synergistic damage to the vascular system since nitrate treatment and diabetes both are associated with oxidative complications. Since PETN is devoid of tolerance [67], oxidative stress [71] and even normalized vascular function in experimental atherosclerosis [134], it might be expected that this nitrate demonstrates beneficial effects in the setting of diabetes. Considering the mechanisms underlying the protective effects of PETN, mainly based on induction of HO-1 [77], ferritin [135], ecSOD [95], and other protective genes/proteins [75] as discussed afore (summarized in Figure 1), PETN indeed could be a suitable nitrate for treatment of ischemia in diabetic patients. Heme oxygenase-1 induction by hemin was also able to suppress nitroglycerin-induced tolerance [77]. Of note are the observations that induction heme oxygenase lead to increased expression of ecSOD [31], and pharmacological as well as genetic overexpression of heme oxygenase protects from diabetic cardiovascular complications [31, 136] (summarized in Figure 1), whereas genetic deficiency in HO-1 aggravates myocardial ischemia/reperfusion injury in diabetic mice [137]. Therefore, it may be speculated that PETN-induced heme oxygenase-1 and ecSOD induction are able to improve vascular function, redox state and even prognosis in diabetic patients. According to an ongoing study in our laboratory, PETN, in contrast to ISMN, is able to improve vascular complications in experimental diabetes. PETN corrected endothelial dysfunction by approximately 50% and improved nitrate resistance almost completely whereas ISMN had only minor effects on vascular dysfunction. Vascular and cardiac mitochondrial ROS formation as well as cardiac NADPH oxidase and serum xanthine oxidase activities were significantly improved by PETN whereas ISMN even increased NADPH oxidase activity. There is also evidence that PETN improves eNOS function and normalizes endothelial dysfunction in experimental diabetes via restoration of tetrahydrobiopterin levels, probably by preventing oxidative depletion of BH_4_ and upregulation of GTP-cyclohydrolase, the BH_4_ synthesizing enzyme. These beneficial effects of PETN on eNOS function, NADPH oxidase activity, and oxidative stress have recently been demonstrated for experimental hypertension in angiotensin-II infused rats [138]. To our best knowledge, this was the first report on the improvement of angiotensin-II-induced hypertension by an organic nitrate.

## 4. Conclusion and Clinical Implications

The endothelium plays a pivotal role in modulating the reactivity of vascular smooth muscle through the formation of several vasoactive substances. There is good evidence for endothelial and smooth muscle dysfunction in diabetes which is shared by the pharmacologically-induced phenomenon of nitrate tolerance. Oxidative stress plays an important role in the setting of both vascular complications and may explain the presence of nitrate resistance in diabetic vessels, a major drawback for the use of nitrates in diabetic patients. Since nitrate tolerance as well as diabetes-associated vascular dysfunction nicely respond to antioxidant treatment, this may be the key to improve the safety and efficacy of a given organic nitrate in diabetic patients. One strategy could be a combination therapy of an antioxidant (e.g., hydralazine or lipoic acid) for which a relief of nitrate tolerance has been previously demonstrated. The combination of ISDN and hydralazine improved the efficacy of the nitrate dramatically in black patients with heart failure. Another promising attempt would be the use of pentaerithrityl tetranitrate which is devoid of nitrate tolerance and oxidative stress due to induction of antioxidant pathways (e.g., HO-1 and ecSOD). It remains to be established whether these highly promising observations, that are mainly based on animal experimental data, may be translated to the clinical situation. First evidence for beneficial effects of PETN treatment in diabetic patients comes from a cost comparison analysis contrasting PETN and ISDN prescribed to diabetic patients in primary care practices in Germany [139]. PETN therapy tended to produce a saving in costs compared to ISDN therapy in diabetic patients when costs for comedication were taken into account and after adjustment for age and comorbidity. Future effort may also be directed towards the development of new organic nitrates that are devoid of tolerance and oxidative stress. A promising strategy is the synthesis of hybrid molecules by introduction of a nitrate function into established cardiovascular drugs such as AT_1_-receptor blockers or glitazones (both drug classes have been demonstrated to prevent the development of nitrate tolerance). These compounds not only could possess intrinsic antioxidant effects, but also provide synergistic antihypertensive, antihyperglycemic, and antiatherosclerotic effects.

##  Glossary

*Clinical nitrate tolerance*: impaired vasodilatory potential of a given organic nitrate in response to chronic use of the drug (may comprise of “pseudotolerance,” “NO resistance,” “endothelial dysfunction” and impaired nitrate bioactivation).

*Nitrate tachyphylaxis*: fast onset of in vitro or in vivo tolerance due to high-dose acute treatment with an organic nitrate (most probably comprises of impaired nitrate bioactivation).

*Primary tolerance*: resistance of the vasculature to endogenous and/or exogenous vasodilators encountered in certain pathophysiological states such as diabetes or coronary artery disease. This primary tolerance may be directed to organic nitrates termed “nitrate resistance” or to other NO donors as well as endogenous NO formation termed “NO resistance.”

*Secondary tolerance*: resistance of the vasculature to exogenous vasodilators encountered after prolonged (clinical tolerance) or high-dose (tachyphylaxis) use of these drugs. This secondary tolerance may be directed to organic nitrates termed “nitrate tolerance” or to other vasodilators generally termed “desensitization.”

*Pseudotolerance:* loss of vasodilatory potential of a given drug due to humoral counterregulation (e.g., activation of the RAAS). Pseudotolerance is closely correlated with the “rebound phenomenon” upon withdrawal of the drug.

*NO resistance*: inadequate vasodilatory response of the vasculature to exogenous or endogenous nitric oxide encountered in certain pathophysiological states such as diabetes or coronary artery disease or upon development of “secondary tolerance.”

*Nitrate resistance*: special form of “primary tolerance.” Inadequate vasodilatory response of the vasculature to exogenous organic nitrate therapy encountered in certain pathophysiological states such as diabetes or coronary artery disease or upon development of “secondary tolerance.”

*Endothelial dysfunction*: inadequate vasodilatory response of the vasculature to exogenous or endogenous endothelium-dependent vasodilators encountered in certain pathophysiological states such as diabetes or coronary artery disease or upon chronic use of organic nitrates (e.g., “cross-tolerance” to acetylcholine).

##  Conflict of Interests 

A. Daiber received vascular research grants and lecture fees from Actavis Deutschland GmbH.

## Abbreviations

ACE: Angiotensin-converting enzyme
ALDH-2: Mitochondrial aldehyde dehydrogenase
ecSOD: Extracellular superoxide dismutase (copper, zinc-containing)
eNOS: Endothelial nitric oxide synthase
HO-1: Heme oxygenase-1
ISMN: Isosorbide-5-mononitrate
ISDN: Isosorbide dinitrate
MnSOD: Manganese superoxide dismutase (mitochondrial isoform)
NO: Nitric oxide (nitrogen monoxide)
PETN: Pentaerithrityl tetranitrate
RAAS: Renin-angiotensin-aldosterone system
ROS: Reactive oxygen species.
